# Improvement of Amniotic Membrane Method for the Treatment of Corneal Perforation

**DOI:** 10.1155/2016/1693815

**Published:** 2016-05-23

**Authors:** Junhua Fan, Meihua Wang, Fulu Zhong

**Affiliations:** Department of Ophthalmology, The 180th Hospital of PLA, Quanzhou, Fujian 362000, China

## Abstract

In our retrospective study we evaluated the efficacy of an improved amniotic membrane (AM) roll-in filling technique (AMR) combined with multilayer amniotic membrane cover to treat corneal perforation and included 46 cornea perforations ≤ 3 mm in diameter treated with AMR and 20% C_3_F_8_ mixed gas filling of the anterior chamber. Anterior chamber depth, aqueous leakage, bubble maintenance time, and cornea morphology were monitored after each operation. The mean diameter of corneal perforation was 1.60 ± 0.55 mm (range 0.5–3) and the success rate of the AMR method for corneal perforation reconstruction was 100% after a single operation. Anterior chamber depth was normally reconstructed without AMR break-off, aqueous leak, or other complications. The mean time of the C_3_F_8_ gas bubble in the anterior chamber was 8.6 ± 2.0 days (range 4–12). At the last follow-up, all patients' visual acuity was improved to varying degrees. The mean follow-up time was 11.0 ± 5.6 months (range 3–36). The AMR plugging combined with multilayer AM cover is a secure and easy intervention, which led to 100% success in our study. Various perforations ranging from trauma to infection can be treated with AMR, which is especially practical in those countries where donor cornea availability is limited.

## 1. Introduction

Corneal perforation is one of the most serious complications of infections and autoimmune diseases as well as traumata. Penetrating keratoplasty (PK) is an effective treatment for corneal perforation [1]. However, in clinical practice, the cornea demand is far greater than the cornea supply. Moreover, although the success rate of corneal allograft transplantation is in general satisfying, infective corneal perforations are still a high-risk during corneal transplantations, because the infective diseases can easily cause corneal graft rejection and infection, eventually leading to graft failure [2].

Amniotic membrane transplantation (AMT) for the treatment of corneal ulcers was first introduced by Lee and Tseng [3] in 1997. Thereafter, it has been widely used for various ocular surface reconstructions. The unique property of amniotic membranes (AMs) in the reconstruction of ocular surfaces is that the AM contains a remarkable mixture of growth factors and cytokines facilitating proliferation and differentiation of epithelial cells, reducing the inflammatory response by inhibiting protease activity, and reducing inflammatory cell activity [4–6]. Therefore, AMT could promote ocular surface tissue healing of persistent epithelial defects (PEDs), corneal ulcers, and eye burns [7–10]. Clinical results showed that the success rate of treatment for corneal ulcers with single or multilayer AMs was over 80% [10, 11], while for cornea perforation with multilayer AMT it was about 73% [12]. Furthermore, the treatment for corneal perforations with fibrin glue-assisted augmented AMT was about 90% [13, 14]. A newly developed AM roll technique has been introduced with a higher success rate for cornea perforation treatments; however, there are few reported treatments in the literature [15, 16]. Based on the usage of AM for healing of cornea tissues and the few reported cases of AMR interventions for cornea perforation treatment, we performed modified AMR interventions combined with multilayer amniotic membrane cover for a variety of corneal perforations up to 3 mm in diameter.

## 2. Patients and Methods

### 2.1. Patients

Between July 2007 and April 2011, 46 eyes of 46 patients (24 men and 22 women with a mean age of 49.4 ± 12.1 years) who suffered from corneal perforations were consecutively admitted to our hospital after failure of conventional ulcer treatment for 5~25 days by different ophthalmologists and treated with AMR filling-in combined with multilayer AM cover. Five cases of corneal perforation (5 eyes) resulted from corneal bacterial infection caused by foreign bodies and 3 cases from spontaneous bacterial infections; in 28 eyes corneal perforations were due to herpes simplex keratitis (HSK), in 2 eyes due to molten iron burns, in 6 eyes due to autoimmune diseases, and in 2 eyes due to fungal keratitis. One of 28 HSK perforations was a biperforation. The time from corneal perforation to receiving surgery was 2–14 days, and the average was 6.2 ± 2.3 days. All patients were treated with ocular antibiotic drops before hospitalization and their visual acuities were all found to be below 20/200. Clinical data, including patient demography, etiology, surgical procedure, associated therapies, visual acuity, and the final outcomes and complications were collected in a retrospective manner (Table 1). Inclusion criterion for our modified treatment was size of perforation ≤3 mm in diameter and exclusion criteria were serious noncorneal diseases such as inflammation of the inner structures of the eyeball (entophthalmia) and severe eyelid defect, which would likely influence corneal recovery. The Ethics Committee of the 180th Hospital of PLA in Quanzhou, China, approved this study and all patients provided written informed consent.

### 2.2. Preparation of Preserved Human AM

Preserved human AMs were obtained from the eye bank of the 180th Hospital of PLA. The preservation method was as previously described [17]. The AMs derived from patients without infectious diseases were peeled from the puerperal placentas and glued to aseptic filter papers, which were cut into 3 × 3 cm pieces. After the epithelial sides of the membranes were treated with 0.5% sodium hyaluronate to protect them from freezing harm, the membrane sheets were stored in anhydrous glycerol at −45°C.

### 2.3. Surgical Procedure

**Figure 1:**
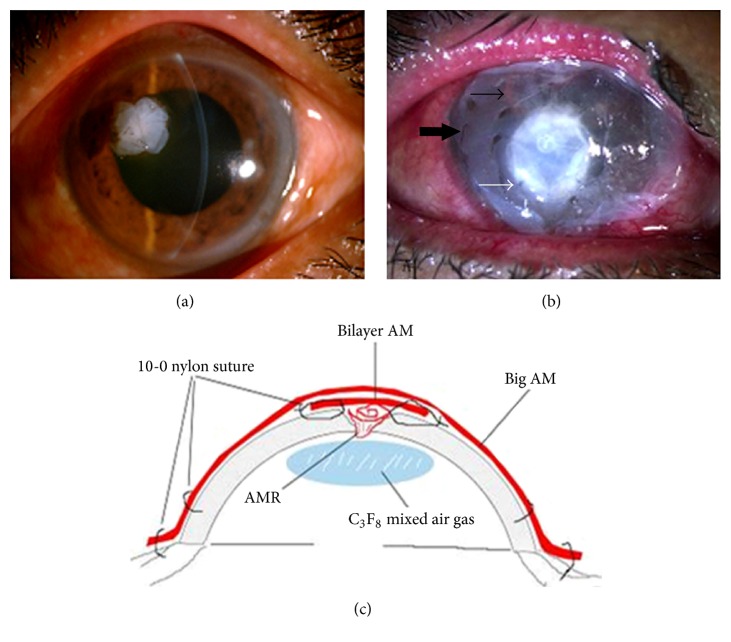
Surgical steps for corneal perforation surgery. For a smaller ulcer and perforation with an abrupt edge, AMR is pushed into the perforation and fixed with cross-stitch fixation as shown in (a). For the larger perforation, first, AM was folded into a roll and plugged into the perforation. Second, a bilayer AM was covered by the roll and ulcer with the epithelial side up and secured with 10-0 nylon sutures (white arrow in (b)). Third, 0.3 mL of 20% C_3_F_8_ (perfluoropropane) was injected into the anterior chamber (thin black arrow in (b)). Finally, a larger piece of AM was applied over the entire cornea as a temporary patch and anchored with 2 laps running 10-0 nylon sutures to the corneal limbus and perilimbal episclera (thick black arrow in (b)). The surgical scheme is shown in (c). Note: the AMR was inserted into the ulcer perforation as a filling material after which the bilayer AM was placed on the ulcer as a cover. Thereafter the big AM was located over the entire cornea with the epithelial side up as an additional cover.

All operations were performed by the same surgeon (Junhua Fan) after patients were anesthetized with 2% lidocaine containing 1 : 8 × 10^4^ epinephrine in the subconjunctival or sub-Tenon's capsule. After local anesthesia, debridement of the ulcer base and necrotic tissue was performed with fine forceps and a microsponge, and the poorly adherent epithelium adjacent to the edge of the ulcer was removed. Afterwards, a little sodium hyaluronate was injected through the perforation into the anterior chamber to separate the synechia between the iris and the cornea. After the frozen AMs were placed in saline for rehydration and thawing for 10 minutes, they were cut into rectangles with dimensions of about 2 × 4 mm and rolled to a length of 2 mm. One end of the roll was plugged into the corneal perforation and the other end was spread over the defective stroma zone (Figure 1(a)).

Based on the size of the perforation, two different ways of fixing the AM rolls were used. For the smaller sized perforation with abrupt edges, we used the suture fixation method, in which the suture needle was inserted into the edge from one side of the cornea hole, penetrated throughout the AM, passed out the edge from the other side of the hole, and then knotted. The depth of the needle insertion was 2/3 of the cornea thickness. If necessary, crisscrossing sewing was used to anchor the AMR to the inside of the hole (Figure 1(b)). For a larger size of perforation, after the AM roll was inserted, a single amniotic membrane was folded in half to become a bilayer amniotic membrane cover. The bilayer amniotic membrane was then trimmed to fit the shape and size of the corneal ulcer. However, caution must be taken to ensure that the outer AM layer was positioned with the epithelial side up because the top of the bilayer AM should serve as a basement membrane for reepithelialization. Then, the edge of the bilayer amniotic membrane was sutured to the healthy corneal tissue around the perforation with a few interrupted stitches using 10-0 nylon suture. Afterwards, a larger piece of AM was applied over the entire cornea as a temporary patch and anchored with 2 flaps using running 10-0 nylon sutures to the corneal limbus and perilimbal episclera (Figure 1(b)). After completion of all sewing, 0.3 mL of 20% C_3_F_8_ (perfluoropropane) mixed with air was slowly injected to separate the iris and the cornea, forming the anterior chamber. It was appropriate to produce a bubble with a diameter of 5-6 mm. After surgery, the conjunctival sac was cleaned with 1/1000 gentamicin solution and then coated with ofloxacin eye ointment; both eyes were bandaged for 2 days; the patients were required to reduce eye movements as much as possible and lie supine on the bed as far as possible to maintain the C_3_F_8_ bubble between the iris and cornea before the bubble was absorbed.  Figure 1(c) shows a scheme of the intervention technique.

### 2.4. Medical Care after Surgery

Postoperatively, antibiotic, sodium hyaluronate, and mydriatic eye drops together with antibiotic eye ointment were instilled. After the bandage was removed, for patients with nonfungal corneal ulcer, 0.5% levofloxacin eye drops (Cravit, Santen Pharmaceutical Company, Osaka, Japan), 0.1% fluorometholone eye drops (Flumetholon, Santen Pharmaceutical Company, Osaka, Japan), 0.1% sodium hyaluronate eye drops (Hyalein 0.1, Santen Pharmaceutical Company, Osaka, Japan), and 0.5% tropicamide eye drops (Shuangxingming, Zhenshiming Pharmaceutical Company, Fuzhou, China) were instilled 4 times a day; 0.3% ofloxacin eye ointment (Dikeluo, Xinqi Pharmaceutical Company, Shenyang, China) was used before sleeping. For patients with fungal corneal ulcer, 0.1% fluorometholone eye drops (Flumetholon, Santen Pharmaceutical Company, Osaka, Japan) were replaced with 5% natamycin eye drops (Natacyn, Alcon Laboratories, Inc.) 6 times a day. We followed up each patient daily in the first week after surgery, weekly for 3 months postoperatively, and randomly at appropriate times. During the follow-ups, visual acuity and intraocular pressure (IOP) were measured and slit-lamp examinations were performed to assess the degree of corneal ulcer healing, graft integrity, signs of inflammation, infection, and changes in the ocular surface and anterior chamber, as well as for monitoring AMR changes. If the temporary big AM graft overlay was found to be loosened or dissolved about 1 week after the operation, we removed it. One month after each operation, morphology was measured at each follow-up with UBM or optical coherence tomography (OCT) to assess the state of AMR fusion and corneal organizations in 33 patients. Surgical success was defined as the cessation of aqueous leakage, an adequate anterior chamber depth, negative result of the Seidel test, complete epithelialization of the AM outermost layer, and formation of a visible stromal thickness at the operated site. Surgical failure was defined as persistence or recurrence of aqueous leakage, lack of epithelialization, or recurrent corneal ulceration.

### 2.5. Statistics

Graphpad Prism 5 was used for statistical analysis. The distribution of each dataset was tested and is presented as the mean ± standard deviation (SD).

## 3. Results

**Figure 2:**
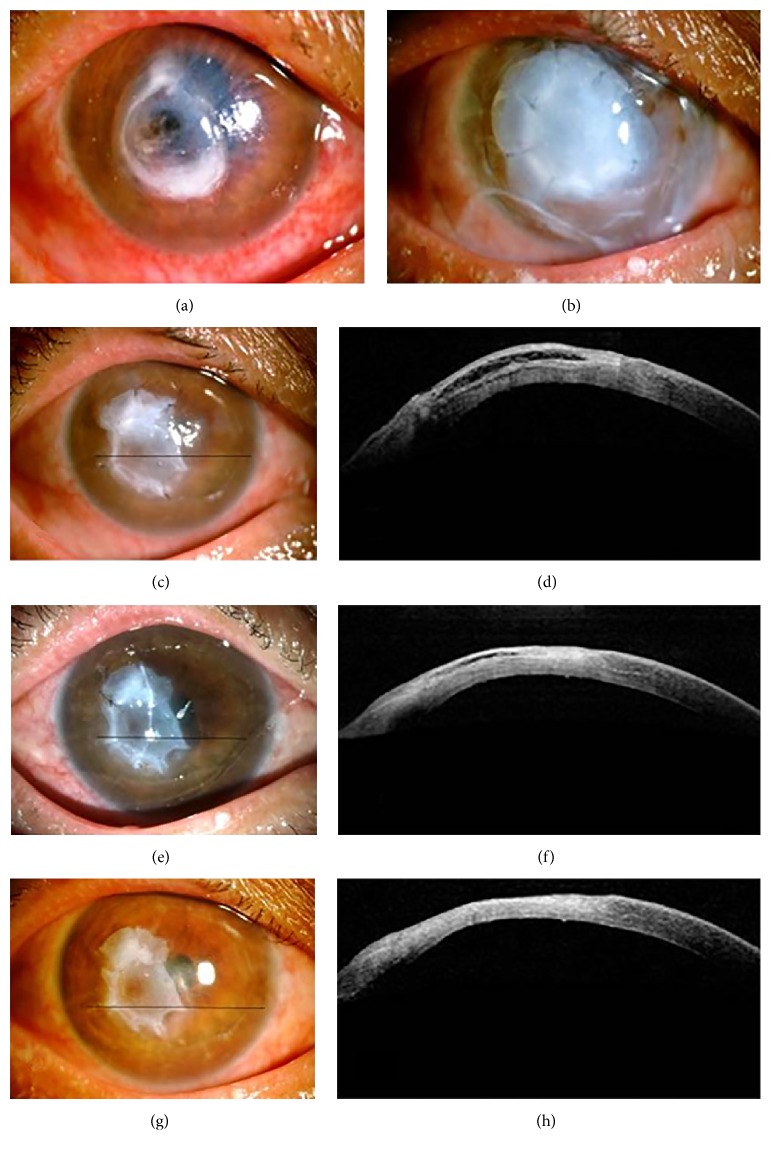
Representative images of a perforation caused by a bacterial corneal ulcer before and after AMR treatment. The black bars in the images on the left indicate the areas shown in the OCT images. (a) A perforation on the cornea before intervention. (b) One week after amniotic membrane transplantation (AMT), the overlay AM still covered the entire cornea. (c) Three weeks after AMT, the overlay AM covering was removed, the bilayer AM was partly dissolved, and the anterior chamber depth was close to normal. (d) OCT image 3 weeks after AMT. The loose layer between the AM and the corneal stroma indicated that the AM was not substituted by corneal collagen tissues yet. (e) Image of the eye 1 month after AMT. (f) OCT scan 1 month after AMT. Most of the loose layer between the AM and the corneal stroma had disappeared with a decrease in the stromal thickness. ((g) and (h)) At 36 days after AMT, the OCT scan indicated tight fusion of the AM and corneal stroma. The stromal thickness at the perforation site was slightly thinner than the surrounding stroma, with hyperreflectivity corresponding to corneal scarring.

The mean diameter of corneal perforation was 1.60 ± 0.55 mm (range 0.5–3), and the mean diameter of the ulcerative stromal defect was 3.77 ± 1.22 mm (range 2–6). The anterior chambers of all patients were flat because of the corneal perforations. The mean time of the C_3_F_8_ gas bubble in the anterior chamber was 8.6 ± 2.0 days (range 4–12). The anterior chamber center depth of all patients recovered to 5 times the corneal thickness, which is above 2.5 mm. One week after surgery, some outermost AM graft cover had begun to loosen or dissolve. After the operation, the average duration of epithelial healing was 1.97 ± 0.56 weeks (range 1–3.5). The average duration of bilayer AM disappearance was 6.89 ± 1.73 weeks (range 4–12) and break after the average fusion time of the amniotic membrane and the corneal stroma was 6.12 ± 1.04 weeks (range 4–8).  Figure 2(c) shows that after the outermost large AM graft was removed, 3 weeks after AMT, the bilayer AM had partly dissolved and the anterior chamber depth was in the normal range. At the same time, the OCT image showed that the AM was fully epithelialized, the perforation was healed completely with an increase in the stromal thickness, and there was a loose layer between the AM and the corneal stroma, indicating that the AM was not substituted by corneal collagen tissues at this time (Figure 2(d)). One month after AMT, the AM and corneal stroma fused together well and most of the loose layer between the AM and the corneal stroma had disappeared with a corresponding decrease in stromal thickness (Figures 2(e) and 2(f)). Thirty-six days after AMT, the AM and corneal stroma fused together tightly and the stromal thickness at the perforation site was slightly thinner than the surrounding stroma, with hyperreflectivity corresponding to corneal scarring (Figures 2(g) and 2(h)).

**Figure 3:**
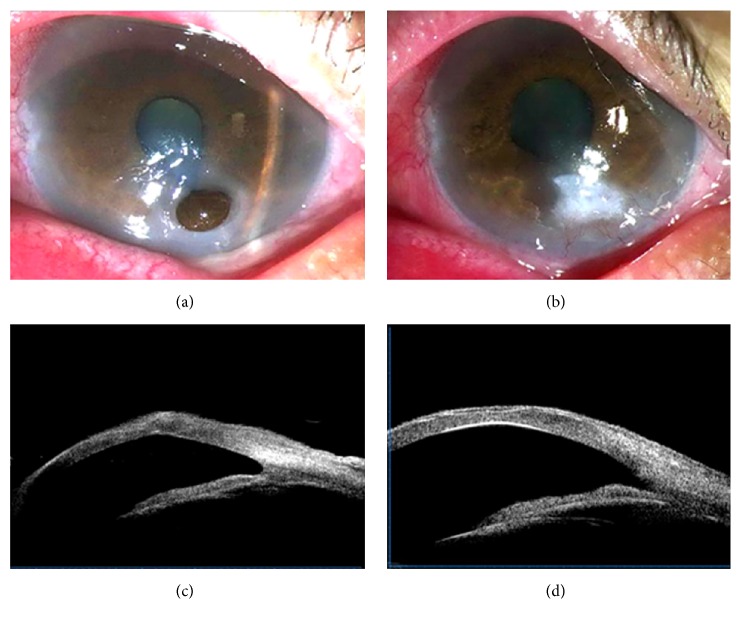
Images of a larger progressive corneal perforation in a patient with recurrent HSK. (a) Perforation before surgery. (b) Image of the same eye 1 month after AMT. (c) UBM image 1 month after AMT showing complete perforation healing. The anterior chamber angle was open and the corneal shape at the perforation site exhibited angulation deformity because of inadequate stromal strength. (d) UBM image 2 months after AMT showing complete cornea reshaping. The front and back surfaces of the cornea were sleek and the stromal thickness at the perforation site was as thick as the surrounding stroma.

Figure 3 shows the healing of a perforation caused by recurrent HSK and measured by UBM.  Figure 3(a) shows the image of an eye before and one month (Figure 3(b)) after AMT in which the corneal perforation was completely healed, the anterior chamber depth was normal, and the AM was fully epithelialized.  Figure 3(c) shows the UBM result 1 month after intervention. The perforation was healed completely, the anterior chamber angle was open, and the corneal shape at the perforation site exhibited angulation deformity because of inadequate stromal strength. However, 2 months after AMT, the reshaping of the cornea was complete and the front and back surfaces of the cornea were sleek, while the stromal thickness at the perforation site was as thick as the surrounding stroma (Figure 3(d)).

The visual acuity at the last follow-up was improved with 6 lines or more in 13 eyes, 3 to 5 lines in 15 eyes, 1 to 2 lines in 14 eyes, and less than 1 line in 4 eyes on the Snellen chart. Three patients whose corneal perforations were located near the corneal limbus had anterior iris synechiae in the areas of the corneal ulcers. From the day after operation, all patients received IOP monitoring with a rebound tonometer (Tianjin Electronic Technology Co., Tianjin, China). One week after the operation, the IOP fluctuation of 46 eyes was at 9~27 mmHg (an average of 15.8 ± 4.4 mmHg); 3 of them showed an IOP higher than 21 mmHg, which recovered to normal levels with treatment of timolol eye drops twice a day for 1 week. The mean time of follow-up was 11.0 ± 5.6 months (range 3–36). No aqueous leakage and pupillary block caused by gas bubbles occurred or secondary infections were detected at any time after surgery, indicating a success rate of 100%. Scars were limited to the ulceration area and as time passed they gradually faded. The results from follow-ups showed that the AMR tamponades did not fall out or fall into the anterior chamber and did not induce any corneal neovascularization. Furthermore, the initial cornea angiogenesis during the cornea inflammation period began to shrink after corneal inflammation and the perforations healed.

## 4. Discussion 

PK is an effective treatment of corneal perforation but because of cultural obstacles in China, donor cornea availability is limited. The treatment effectiveness of amniotic membrane overlaying a corneal ulcer has been verified. However, for a deeper corneal ulcer and even corneal perforation, a single or multilayer amniotic membrane overlay cannot plug the defective zone. Moreover, surface reconstruction should include filling of new collagen into the corneal ulcer bed, thus promoting new basement membrane formation for rapid epithelialization and suppressing inflammation. In the present study, the amniotic membrane was rolled and then plugged into the defect zone of the cornea; then a bilayer amniotic membrane was used to overlay and fix the amniotic roll, and finally a larger amniotic membrane was used to cover the whole cornea. As a result, enough force was generated to resist the pressure of the anterior chamber and to prevent any aqueous leakage and outward bulging of the AMR during the postoperative period. In addition to filling the defective corneal area, it provided an ideal basement for rapid epithelialization and wound healing. Furthermore, the large AM covering the whole cornea constitutes a mechanical protection for the fragile corneal epithelium, allowing sufficient oxygenation and hydration of the epithelial cells and suppressing inflammation [18]. In the present study, ocular surface inflammation was markedly reduced after AMT, in agreement with previously published reports [12, 13]. It is noteworthy that AM contains a mixture of growth factors, neurotrophins and cytokines that facilitate proliferation and differentiation of epithelial cells, and reduces the inflammatory response [5, 19, 20]. Additionally, AM can be also used to prevent collagenous tissue being uncovered and collagenolysis.

When amniotic roll plugging was employed, the aqueous fluid could easily ooze along the amniotic membrane roll due to the anterior chamber pressure resulting in disappearance of the anterior chamber. To solve this problem, we used a gas-solution interfacial tension to prevent aqueous oozing along the AMR. First, we tried to inject sterile air bubbles into the anterior chamber but two days later they disappeared due to absorption, and aqueous fluid again oozed along the AMR. Therefore, we changed to a C_3_F_8_ gas mixture injection, which lasted a relatively long time (more than 1 week) [21]. As a result, aqueous fluid oozing was prevented, the depth of anterior chamber was maintained, and anterior synechia was prevented.

The technique used in the current study was not practiced for corneal perforation sizes ≥3 mm in diameter, because we were worried that there might be a higher risk of using the method for larger cornea perforation sizes. In general, the larger the size of the corneal perforation, the higher the pressure of aqueous humor on the amniotic roll, which might lead to complications such as aqueous leakage, hydrops under the amniotic membrane, and outward bulging of the AM [12].

Special attention during the operation must be paid to fine suturing of the double amniotic membrane film surface, because if the stitches are too large (>1 mm), when injecting gas to the anterior chamber after the operation, the amniotic roll may be extruded from the suture by anterior chamber pressure. Also too large bubble in the anterior chamber should be avoided, because it may cause amniotic roll bulging and temporary high intraocular pressure after the operation. For larger ulcer surfaces and perforated ulcers with a thin basement, if the amniotic roll is packed too hard it may cause holes to expand or tear and amniotic roll luxation into the anterior chamber and the intervention must be redone as PK.

However, for lesions ≤3 mm our study showed in the longer term follow-ups that 2 months after intervention the results of UBM or anterior segment OCT (AS-OCT) examinations revealed the successful fusion of AM rolls with corneal tissues which filled the lesion areas. Finally, the AM rolls were replaced with corneal scar tissue and the perforation areas were recovered to the thickness of normal corneas with both well-shaped corneal sides becoming mellow.

In summary, under conditions when corneal transplantations cannot be performed due to a lack of fresh donor corneas, AMR combined with the multilayer amniotic membrane cover method is an effective alternative to conjunctival transplantations or conjunctival flap covering surgeries, with a success rate of 100%. The eyesight partly recovered after surgery, no neovascularization was induced, and the technique was acceptable in terms of cosmesis. Unnecessary trauma at the donor site can be avoided compared to conjunctival flap overlaying. Furthermore, our method might also provide sufficient time to obtain a fresh donor cornea and a good condition for future successful corneal transplantation, thereby converting emergency tectonic PKP into elective optical PKP, which is a more favorable procedure with a better outcome for the patient's vision. However, long-term studies with larger samples of patients with various types of perforations are still needed.

## Figures and Tables

**Table 1:** Pre- and postoperative records of 46 patients with cornea perforations.

Case/age/sex	Underlying etiology	Other complications	Max. perforation diameter (mm)	Max. ulcer diameter (mm)	Preoperative VA	VA at last follow-up	C_3_F_8_ gas remaining (days)	IOP 1 week postoperatively (mmHg)	Last follow-up (months)
1/22/M	Neurotrophic, HSK; CNV	No	Double perforation 0.5/1.0	0.5/1.0	HM	CF	5	14	3
2/35/M	Molten iron burn, IU	No	1	2.5	CF	20/100	8	19	18
3/45/F	AD, RA	No	1.5	3	20/200	20/40	9	18	11
4/51/M	Neurotrophic, HSK	DM	1	2.5	CF	20/200	12	21	12
5/54/F	Neurotrophic, post-HSK, CNV	Dry eye	1.5	2.5	HM	20/150	10	18	12
6/53/F	Neurotrophic, HSK	Dry eye	2	4.0	HM	20/200	7	16	6
7/46/M	Neurotrophic, HSK	No	1.5	3.5	HM	20/180	8	20	18
8/37/M	FB, IU	No	2	4.0	HM	20/200	6	14	6
9/64/F	Neurotrophic, post-HSK	Dry eye	1.5	5.0	HM	20/180	5	15	10
10/62/F	Neurotrophic, post-HSK	HT	1.5	4.0	HM	20/135	4	13	12
11/36/F	AD, RA	No	2	5	20/200	20/80	7	12	15
12/42/M	FB, IU	No	1.5	3	CF	20/30	8	20	12
13/59/M	Neurotrophic, post-HSK, CNV	Dry eye	2	5	LP	CF	9	27	13
14/40/M	AD, RA	No	2	6	HM	20/85	10	17	14
15/58/M	Neurotrophic, post-HSK	DM	2	5.5	LP	20/200	11	18	4
16/46/F	SBI	HT	3	6	LP	20/200	10	13	6
17/76/M	SBI	HT	2.5	5.5	HM	20/130	8	10	36
18/31/F	HSK	No	1.5	2.5	20/200	20/100	6	20	8
19/44/M	HSK, CNV	No	1	3	CF	20/80	12	21	10
20/56/F	Neurotrophic, post-HSK	No	1.5	4	20/200	20/35	7	14	12
21/38/F	AD, RA	No	2	5	CF	20/80	8	19	6
22/62/F	FK	DM	1.5	5	HM	20/170	9	12	12
23/47/M	HSK	Dry eye	1	3	CF	20/100	10	11	14
24/57/F	Neurotrophic, post-HSK	Dry eye	2	4.5	HM	20/200	11	16	12
25/48/M	FB, IU	No	1.5	3.5	HM	20/80	8	11	8
26/54/F	Neurotrophic, post-HSK, CNV	No	1	4.0	LP	20/30	7	10	24
27/36/F	HSK	No	1.5	2.5	CF	20/25	8	20	14
28/61/M	FB, IU	No	2	4	HM	20/120	11	14	10
29/53/M	Neurotrophic, post-HSK, IU, CNV	No	1.5	5.5	LP	20/250	12	9	10
30/55/M	AD	Mooren's ulcer	2	6.0	20/200	20/40	8	26	6
31/62/F	SBI	HT	3	5.5	LP	20/400	9	24	6
32/45/M	HSK, CNV	No	1	2.0	HM	20/100	10	15	10
33/44/F	HSK	No	1.5	1.5	CF	20/30	11	10	10
34/57/M	Neurotrophic, post-HSK	No	1.5	3.0	CF	20/150	5	19	12
35/65/F	SBI	HT	2	5.0	HM	20/160	9	13	8
36/51/F	Neurotrophic, post-HSK, CNV	No	1.5	2.5	HM	20/40	10	12	12
37/39/M	FB, IU	No	1	3.0	CF	20/40	8	11	8
38/45/F	FB	No	0.5	1.5	CF	20/25	10	20	4
39/52/F	HSK	No	1	3.0	CF	20/50	11	14	9
40/59/M	Neurotrophic, post-HSK, CNV	No	1.5	4.0	HM	20/100	9	19	6
41/55/M	Neurotrophic, post-HSK, CNV	Dry eye	1.5	3.5	HM	20/200	8	14	10
42/63/F	FK	HT	2.5	5.5	HM	20/160	7	15	12
43/57/F	AD, RA	No	2	6.0	CF	20/80	11	18	18
44/28/M	HSK	No	1	2.5	20/200	20/20	6	11	6
45/30/M	Molten iron burn, IU	No	1.5	4.0	HM	20/130	9	10	10
46/50/M	HSK, CNV	No	1	3.5	20/300	20/40	8	14	12

AD = autoimmune diseases; CF = counting fingers; CNV = corneal neovascularization; DM = diabetes mellitus; FB = foreign body; FK = fungal keratitis; HM = hand motion; HP = hypopyon; HSK = herpes simplex keratitis; HT = hypertension; IOP = intraocular pressure; IU = infectious ulcer; LP = light perception; RA = rheumatoid arthritis; SBI = spontaneous bacterial infection; VA = visual acuity.
